# Pivotal Role of IL-22 Binding Protein in the Epithelial Autoregulation of Interleukin-22 Signaling in the Control of Skin Inflammation

**DOI:** 10.3389/fimmu.2018.01418

**Published:** 2018-06-21

**Authors:** Tomohiro Fukaya, Takehito Fukui, Tomofumi Uto, Hideaki Takagi, Junta Nasu, Noriaki Miyanaga, Keiichi Arimura, Takeshi Nakamura, Haruhiko Koseki, Narantsog Choijookhuu, Yoshitaka Hishikawa, Katsuaki Sato

**Affiliations:** ^1^Division of Immunology, Department of Infectious Diseases, Faculty of Medicine, University of Miyazaki, Miyazaki, Japan; ^2^Japan Agency for Medical Research and Development (AMED), Tokyo, Japan; ^3^Department of Oral and Maxillofacial Surgery, Faculty of Medicine, University of Miyazaki, Miyazaki, Japan; ^4^Department of Otolaryngology, Head and Neck Surgery, Faculty of Medicine, University of Miyazaki, Miyazaki, Japan; ^5^Laboratory for Developmental Genetics, RIKEN Center for Integrative Medical Sciences, Yokohama, Japan; ^6^Division of Histochemistry and Cell Biology, Department of Anatomy, Faculty of Medicine, University of Miyazaki, Miyazaki, Japan

**Keywords:** interleukin-22, interleukin-22 binding protein, autoregulation, epidermal keratinocytes, skin inflammation, psoriasiform dermatitis, effector T cells

## Abstract

Disruption of skin homeostasis can lead to inflammatory cutaneous diseases resulting from the dysregulated interplay between epithelial keratinocytes and immune cells. Interleukin (IL)-22 signaling through membrane-bound IL-22 receptor 1 (IL-22R1) is crucial to maintain cutaneous epithelial integrity, and its malfunction mediates deleterious skin inflammation. While IL-22 binding protein (IL-22BP) binds IL-22 to suppress IL-22 signaling, how IL-22BP controls epithelial functionality to prevent skin inflammation remains unclear. Here, we describe the pivotal role of IL-22BP in mediating epithelial autoregulation of IL-22 signaling for the control of cutaneous pathogenesis. Unlike prominent expression of IL-22BP in dendritic cells in lymphoid tissues, epidermal keratinocytes predominantly expressed IL-22BP in the skin in the steady state, whereas its expression decreased during the development of psoriatic inflammation. Deficiency in IL-22BP aggravates psoriasiform dermatitis, accompanied by abnormal hyperproliferation of keratinocytes and excessive cutaneous inflammation as well as enhanced dermal infiltration of granulocytes and γδT cells. Furthermore, IL-22BP abrogates the functional alternations of keratinocytes upon stimulation with IL-22. On the other hand, treatment with IL-22BP alleviates the severity of cutaneous pathology and inflammation in psoriatic mice. Thus, the fine-tuning of IL-22 signaling through autocrine IL-22BP production in keratinocytes is instrumental in the maintenance of skin homeostasis.

## Introduction

The maintenance of structural and immunological barrier function in the skin is a prerequisite for limiting exposure to potentially harmful environmental substances and preventing systemic dissemination and entry of commensal and pathogenic microorganisms (1, 2). Therefore, tight regulation for crosstalk between epithelial, mesenchymal, and immune cells is essential to achieve effective host defense, maintain a healthy skin homeostasis, and allow normal wound repair after injury (1, 2). However, disruption of skin homeostasis is thought to cause chronic inflammatory skin diseases resulting from the dysregulated interplay between epithelial keratinocytes and infiltrating inflammatory leukocytes (1–3).

Interleukin (IL)-22 is a member of the IL-10 cytokine family with multiple functions in various inflammatory responses, depending on the environmental context. IL-22 is mainly produced by CD4^+^ effector T (Teff) cells, including T helper 1 (Th1) cells, Th17 cells, and Th22 cells, as well as innate lymphoid cells (ILCs) 3 (4–6). IL-22 signals through a heterodimeric transmembrane receptor complex composed of IL-22 receptor 1 (IL-22R1, also known as IL-22RA1) and IL-10 receptor 2 (IL-10R2), whereas IL-10 signals through IL-10R1 and IL-10R2 (4–6). In contrast to the ubiquitous expression of IL-10R2 on immune cells, the expression of IL-22R1 is restricted to non-hematopoietic cells at body barriers including epithelial cells of the lung and gastrointestinal tract and keratinocytes in the skin, thereby allowing IL-22 to be an important cytokine mediating the crosstalk between leukocytes and epithelia at barrier surfaces (4–6). Similar to other members of the IL-10 family, IL-22 mediates its effects through the IL-22R1/IL-10R2 complex and subsequent JAK–signal transducer and activator of transcription (STAT) signaling pathways, including Jak1, Tyk2, and STAT3 (6). As the main physiological function of IL-22 includes a reinforcement of epithelium barrier function through induction of antimicrobial peptides (AMPs) to promote antimicrobial immunity (7), and wound healing through induction of epithelial cell proliferation and survival following tissue damage in pathophysiological conditions (8–10), it appears to have an important role in the maintenance of epithelial integrity at barrier surfaces (11, 12). Nevertheless, continuous exacerbated or uncontrolled IL-22 signaling leads to undesirable tissue inflammation, accelerating certain immune pathologies such as psoriasis and rheumatoid arthritis (6, 13, 14), probably depending on the inflammatory milieu through synergistic action with other proinflammatory cytokines. Therefore, the fine-tuning of IL-22 signaling is implicated to be required to maintain epithelial integrity and control cutaneous tissue inflammation and immunosurveillance, as well as normal skin wound healing.

Unlike other members of the IL-10 cytokine family, IL-22 has a soluble-secreted receptor, known as IL-22 binding protein (IL-22BP, also known as IL-22RA2) (6, 15–17). Although IL-22BP shares the highest structural homology with the IL-22R1 chain, IL-22BP exhibits a much higher affinity for IL-22 than IL-22R1 and therefore prevents the binding of IL-22 to IL-22R1 (18–20). While the constitutive expression of IL-22BP has been reportedly observed in several tissues at various levels, subsets of CD11b^+^ conventional dendritic cells (cDCs) are a major source of IL-22BP in the gastrointestinal tract (21), and appear to be involved in regulation of intestinal tissue repair and tumorigenesis in the colon through the suppression of IL-22 function (22). Furthermore, IL-22BP expressed by CD11b^+^ cDCs has been shown to promote the bacterial uptake into Payer’s patch (PP) by inhibiting IL-22 signaling in the follicle-associated epithelium (23). On the other hand, IL-22BP has reportedly mediated a pathogenic role of intestinal CD4^+^ T cells in inflammatory bowel disease through impairment of the protective action of IL-22 (24). However, despite data linking the imbalance in IL-22–IL-22BP axis to psoriatic dermatitis (14, 25), intrinsic cutaneous cellular sources of IL-22BP and the role of endogenous IL-22BP in the functionality of keratinocytes that impact skin pathologic inflammation remain unclear.

Here, we address how IL-22BP controls epithelial functionality to prevent skin inflammation. We provide evidence that IL-22BP is predominantly expressed in keratinocytes in the skin that are different from other tissues, suppressing their IL-22-mediated functional alternations. We further demonstrate that deficiency of IL-22BP promotes the progression of psoriasiform dermatitis mediated through enhanced epidermal hyperplasia and inflammation. Collectively, these findings indicate that IL-22BP mediates epithelial autoregulation of IL-22 signaling to control cutaneous pathogenesis.

## Materials and Methods

### Mice

The following 8- to 12-week-old mice were used in this study. C57BL/6 mice (Japan Clea), B6.FVB-Tg*^Itgax-DTR/EGFP^* Lan/J (CD11c-DTR/EGFP) mice (26) (referred to as cDC-ablated mice), and B6.*Il22ra2^−/−^* mice as described below. All mice were bred and maintained in SPF and germ-free (GF) conditions in the animal facility at the University of Miyazaki. All experiments were performed in accordance with institutional guidelines of the Animal Experiment Committee and Gene Recombination Experiment Committee. For the systemic ablation of cDCs, B6.CD11c-DTR/EGFP mice were intraperitoneally (i.p.) injected with DT (100 ng/mouse; Sigma-Aldrich), and B6.WT mice were also treated with DT as controls.

### Additional Methods

Generation of *Il22ra2^−/−^* mice, tissue and cell isolation, flow cytometry, quantitative RT-PCR, generation of human immunoglobulin G Fc fusion protein, *in vitro* CD4^+^ T-cell differentiation assay, cell culture, skin inflammation, histopathological assessment, and immunohistochemical analysis are described in Supplementary Material.

### Statistical Analysis

Data are expressed as the mean ± SD from 3 to 10 individual samples in a single experiment, and we performed at least three independent experiments. The statistical significance of the differences between the values obtained was evaluated by ANOVA. A *P* value of <0.01 was considered significant.

## Results

### IL-22BP Is Predominantly Expressed by Keratinocytes in the Skin

Previous studies have shown that the *Il22ra2* transcript is preferentially expressed in secondary lymphoid tissues and intestinal tissues compared with other tissues, and its expression is mainly detected in cDCs (21–23). However, the expression level and cellular source of IL-22BP in the skin relative to other lymphoid and non-lymphoid tissues remain unknown. To understand the role of endogenous IL-22BP in the control of skin homeostasis, we first compared the transcriptional expression levels of *Il22ra2*, as well as *Il22*, throughout lymphoid and non-lymphoid tissues, including epithelial tissues of the skin, in the steady-state condition. Similar to the published reports (5, 6), high transcriptional expressions of *Il22* and *Il22ra2* were found in peripheral lymph nodes (PLNs) and mesenteric lymph nodes (MLNs), as well as colon and PP (Figures S1A,B in Supplementary Material), and CD11c^high^I-A/I-E^+^ cDCs showed a higher expression of the *Il22ra2* transcript than other leukocytes in both PLNs and MLNs, but not in the spleen (Spl) (Figures S1C–E in Supplementary Material). On the other hand, the marked expression level of the *Il22ra2* transcript in the ears and back skin was comparable to that of PP, whereas little or no transcriptional expression of the *Il22* was observed in the skin (Figures S1A,B in Supplementary Material). Furthermore, the prominent expression of *Il22ra2* transcript was detected in CD45^−^ epidermal keratinocytes and CD45^−^ dermal mesenchymal cells rather than CD45^+^I-A/I-E^−^ leukocytes and CD45^+^I-A/I-E^+^ leukocytes in the epidermis and dermis, respectively (Figures S1F,G in Supplementary Material).

To confirm that cutaneous epithelial cells, but not CD11c^high^I-A/I-E^+^ cDCs, act as the main producer of IL-22BP, we examined the transcriptional expression of *Il22ra2* in the absence of cDCs using CD11c-diphtheria toxin (DT) receptor (DTR)/enhanced green fluorescent protein (EGFP) transgenic (Tg) mice (26), in which CD11c^high^EGFP^+^ cDCs were depleted after a single DT injection (referred to as cDC-ablated mice). The depletion of cDCs enhanced the expression levels of the *Il22ra2* transcript in Spl possibly due to the massive splenic infiltration of CD11b^+^Gr-1^+^ neutrophils (27, 28) with their high expression after DT treatment (Figures S2A–C in Supplementary Material). By contrast, the elimination of cDCs led to a reduction in the transcriptional expression of *Il22ra2* in PLNs, MLNs, and PP (Figure S2A in Supplementary Material), suggesting that cDCs mainly produce IL-22BP in these secondary lymphoid tissues. However, the cutaneous expression level of the *Il22ra2* transcript was not affected the absence of cDCs (Figure S2A in Supplementary Material).

Collectively, these results indicate that epidermal keratinocytes are the major cellular source of IL-22BP in the skin in the steady-state condition.

To clarify the kinetics for the expression of IL-22BP during the skin inflammation, we accessed the expression level of the *Il22ra2* transcript in the skin and skin-draining PLNs in psoriasiform dermatitis induced by topical application of imiquimod (IMQ), known as a synthetic Toll-like receptor 7 (TLR7) ligand on the ear skin (29, 30). The transcriptional expression of *Il22ra2* was decreased during the development of psoriasiform dermatitis in ear skin and skin-draining PLNs (Figures S1H,I in Supplementary Material).

As retinoic acid (RA) enhanced the transcriptional expression of *Il22ra2* on human monocyte-derived DCs (21), we examined the influence of topical application of RA on the expression of the *Il22ra2* transcript in keratinocytes. In contrast to the enhanced transcriptional expression of *Il22ra2* in bone marrow (BM)-derived cDCs upon stimulation with RA, reduced expression of the *Il22ra2* transcript was observed in keratinocytes after treatment with RA (Figures S2D,E in Supplementary Material). Indeed, the topical application of RA reduced the transcriptional expression of *Il22ra2* in ear skin (Figure S2F in Supplementary Material).

It has been shown that the production of IL-22 and bacterial colonization is reciprocally regulated in the mucosal surface of the intestine (23, 31, 32). To address the influence of bacterial colonization on the production of IL-22 and IL-22BP, we compared their transcriptional expressions in the skin between specific pathogen-free (SPF) and GF wild-type (WT) mice. While the cutaneous expression of the *Il22* transcript was barely detected in GF mice when compared with that of SPF mice, comparable expression of the *Il22ra2* transcript was observed in the ear skin in GF and SPF mice (Figure S2G in Supplementary Material).

### Deficiency of IL-22BP Aggravates Skin Inflammation

**Figure 1:**
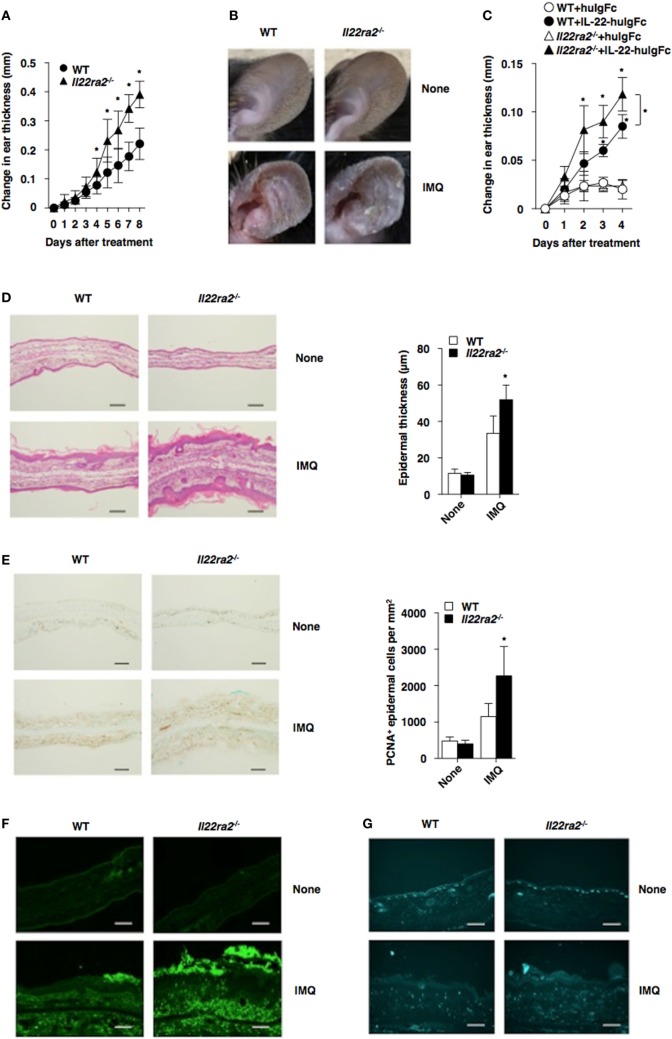
IL-22 binding protein deficiency exacerbates psoriatic dermatitis. **(A,B)** Wild-type (WT) mice (*n* = 5) and *Il22ra2^−/−^* mice (*n* = 5) were treated topically with imiquimod (IMQ) on the left ear skin every day for 8 days. **(A)** Ear thickness was evaluated for 8 days. Data were obtained from five individual samples in a single experiment. *P < 0.01 compared with WT mice. **(B)** Representative pictures of ear skin lesions at 8 days. **(C)** WT mice (*n* = 5) and *Il22ra2^−/−^* mice (*n* = 5) were treated with or without Fc fragment of human immunoglobulin (huIgFc) or interleukin (IL)-22-huIgFc, and ear thickness was evaluated for 4 days. Data were obtained from five individual samples in a single experiment. *P < 0.01 compared with WT mice. **(D–G)** Psoriatic dermatitis was induced by the topical application with IMQ on the left ear of WT mice (*n* = 5) and *Il22ra2^−/−^* mice (*n* = 5). **(D)** Representative hematoxylin and eosin sections (magnification; 20×) of ear skin at days 0 and 8 (left panel), and epidermal thickness was evaluated at 8 days (right panel). Data were obtained from five individual samples in a single experiment. *P < 0.01 compared with WT mice. **(E)** Representative immunohistochemical sections for detecting proliferating cell nuclear antigen (PCNA) (magnification; 20×) of ear skin at days 0 and 8 (left panel), and the quantification of PCNA^+^ epidermal cells of the epidermis (right panel) at 8 days. Data were obtained from five individual samples in a single experiment. *P < 0.01 compared with WT mice. **(F,G)** Representative immunohistochemical sections (magnification; 20×) for detecting Gr-1 **(F)** and γδTCR **(G)** of ear skin at days 0 and 8. All data are representative of at least three independent experiments.

Accumulating evidence indicates that IL-22 mediates keratinocyte proliferation and diffuse epidermal hyperplasia that cause thickening of the epidermis known as acanthosis, inhibits terminal differentiation of keratinocytes leading to incomplete keratinization that causes parakeratosis, and induces the secretion of AMPs (14, 25). To evaluate the pathophysiological role of IL-22BP *in vivo*, we generated mice lacking the *Il22ra2* gene (*Il22ra2*^−/−^; Figure S3 in Supplementary Material). *Il22ra2^−/−^* mice were born at the expected frequencies, and homozygous mice were healthy with normal cellularity in primary and secondary lymphoid tissues (Figure S4 in Supplementary Material). Upon topical application of IMQ on the ear skin, *Il22ra2^−/−^* mice exhibited a more prominent psoriasiform inflammation, with thickening and scaling than WT mice (Figures 1A,B). Furthermore, the intradermal (i.d.) injection of IL-22 fusion protein, which consists of IL-22 and the Fc fragment of human immunoglobulin G (huIgG) (IL-22-huIgFc), caused more severe psoriatic inflammation in *Il22ra2^−/−^* mice than WT mice (Figure 1C), suggesting that the dysregulation of IL-22 signaling mediates the progression of psoriatic inflammation. Histological analyses confirmed that *Il22ra2^−/−^* mice displayed a more significant parakeratosis, acanthosis, and Munro’s microabscesses, as well as cutaneous infiltration of mononuclear cells, including Gr-1^+^ granulocytes and γδTCR^+^ T cells than WT mice following topical application of IMQ (Figures 1D–G).

**Figure 2:**
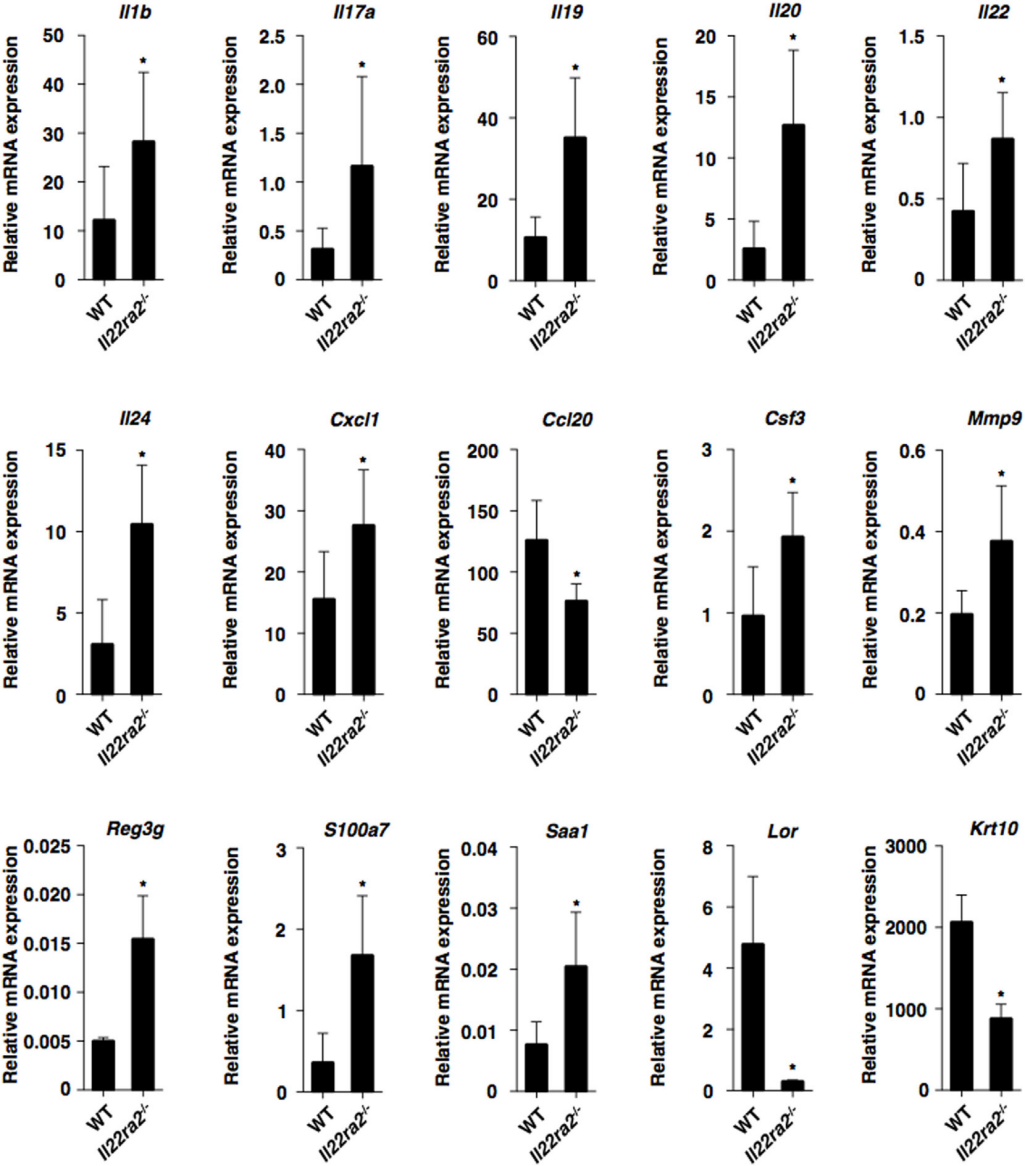
IL-22 binding protein deficiency promotes psoriatic inflammation. Transcriptional expression of cytokines, chemokines, and epithelial inflammation-related molecules in ear skin was analyzed by quantitative reverse transcription polymerase chain reaction at 8 days after topical application of imiquimod on the ear skin in wild-type (WT) mice (*n* = 5) and *Il22ra2^−/−^* mice (*n* = 5), and expression was normalized to the *Actb* transcript. Data are the mean ± SD from three individual samples in a single experiment. *P < 0.01 compared with WT mice. All data are representative of at least three independent experiments.

To clarify how IL-22BP controls psoriasiform inflammation, we compared the transcriptional expressions of cytokines and chemokines, as well as epithelial inflammation-related molecules, in psoriatic lesions between WT mice and *Il22ra2^−/−^* mice (Figure 2; Figure S5A in Supplementary Material). *Il22ra2^−/−^* mice showed higher transcriptional expression of *Il17a*, I*l22, Il1b, Csf3, Cxcl1, Mmp9, Il19, Il20, Il24, Reg3g* (33), *S100a7*, and *Saa1* (34–36) than WT mice. On the other hand, they exhibited lower transcriptional expressions of *Cc120* (14), *Lor*, and Krt10 which are known as terminal differentiation marker genes (33), than WT mice.

**Figure 3:**
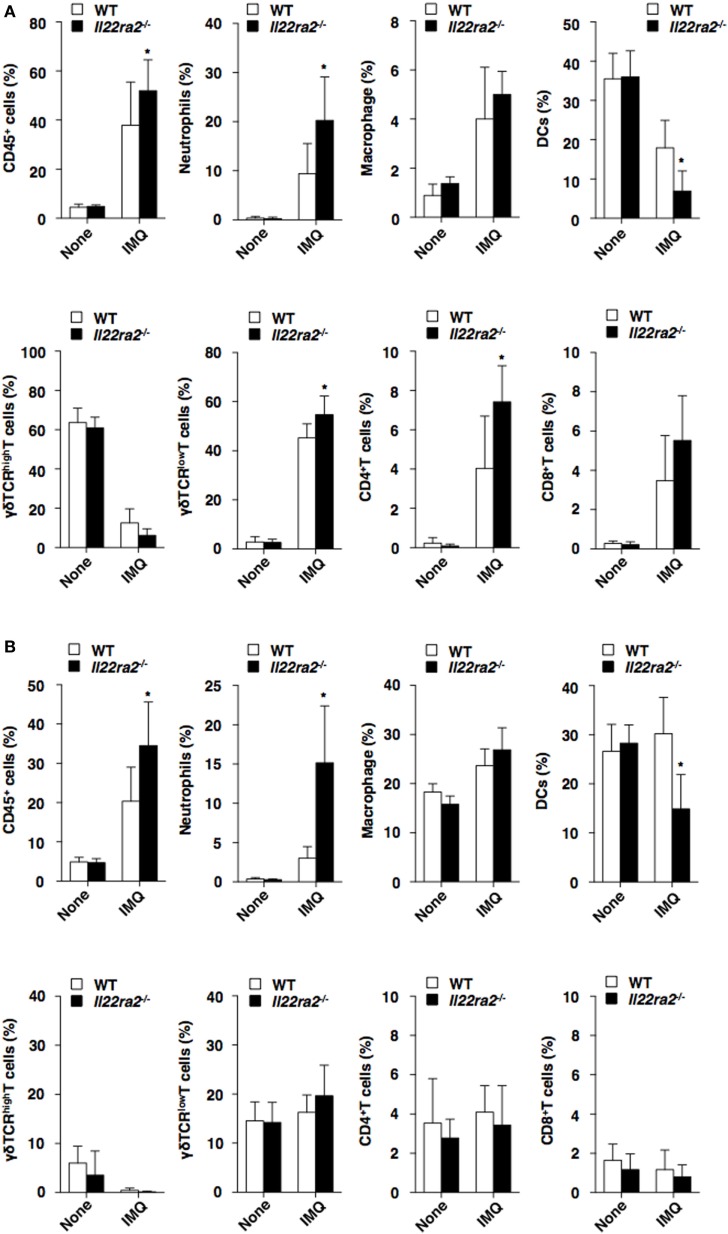
Absence of IL-22 binding protein enhances the accumulation of inflammatory leukocytes in psoriatic skin. The frequency of leukocytes in the epidermis **(A)** and dermis **(B)** was analyzed by flow cytometry at days 0 and 8 after topical application of imiquimod (IMQ) on the ear skin in wild-type (WT) mice (*n* = 5) and *Il22ra2^−/−^* mice (*n* = 5). Data are the mean ± SD from three individual samples in a single experiment. *P < 0.01 compared with WT mice. All data are representative of at least three independent experiments.

We also assessed the impact of IL-22BP on the infiltration of inflammatory leukocytes in the skin following initiation of psoriasiform dermatitis. *Il22ra2^−/−^* mice showed enhanced or reduced epidermal infiltration of neutrophils and γδTCR^low^ T cells, as well as CD4^+^ T cells or DCs, whereas they exhibited enhanced or reduced dermal infiltration of neutrophils or DCs when compared with WT mice (Figure 3). Furthermore, *Il22ra2^−/−^* mice showed higher accumulation of neutrophils, macrophages, cDCs, plasmacytoid DCs, and γδTCR^+^ T cells in the skin-draining PLNs than WT mice (Figure S5B in Supplementary Material).

Taken together, these results indicate that a deficiency in IL-22BP exacerbates skin inflammation.

### IL-22BP Abrogates the IL-22-Mediated Alternation of Epithelial Functionality

**Figure 4:**
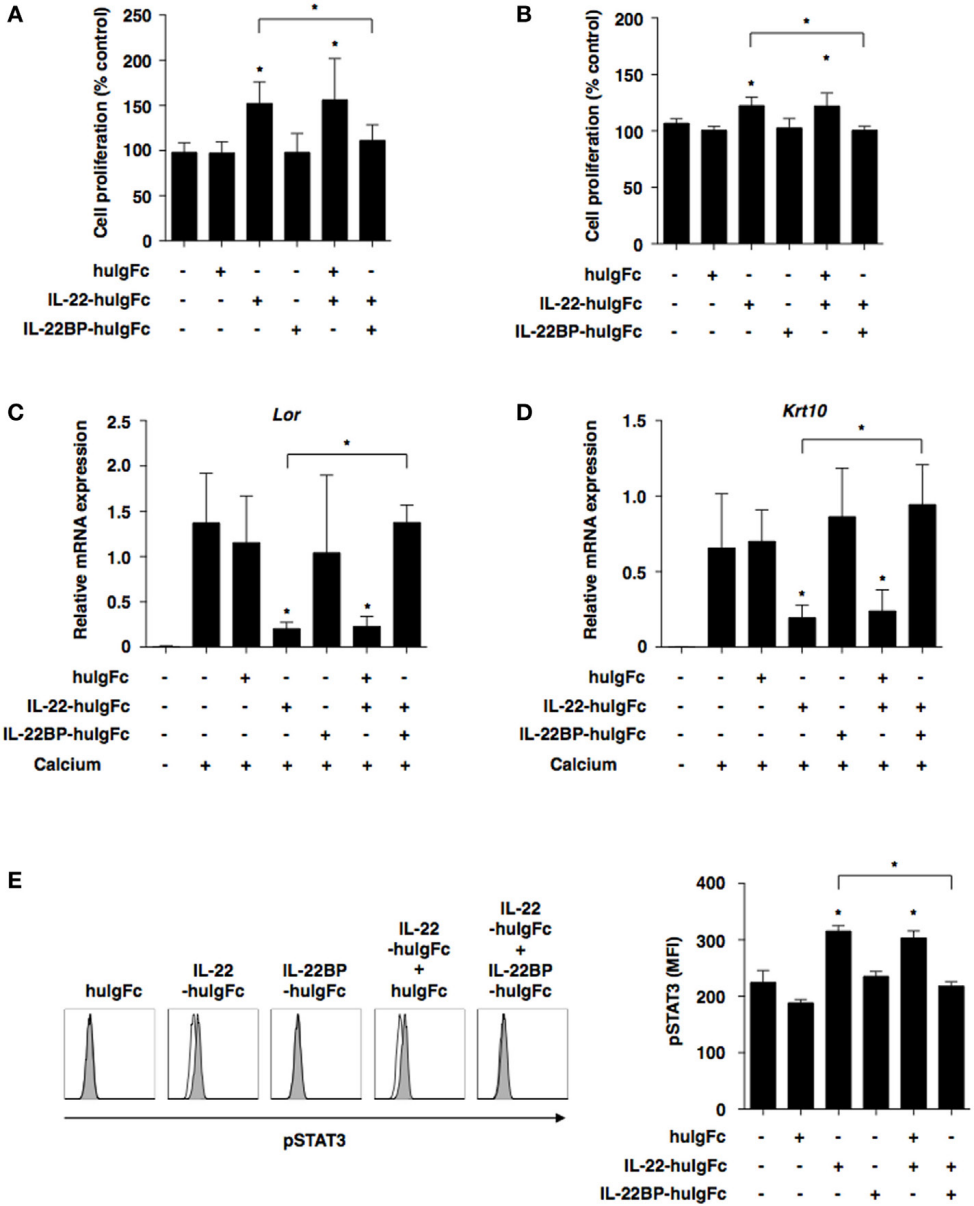
IL-22 binding protein (IL-22BP) suppresses the interleukin (IL)-22-mediated functional alternation of keratinocytes. **(A–D)** Keratinocytes were cultured with or without Fc fragment of human immunoglobulin (huIgFc), IL-22-huIgFc, and/or IL-22BP-huIgFc for 3 days. **(A,B)** Proliferative responses of keratinocytes in the absence **(A)** or presence **(B)** of extracellular calcium, with data expressed as percentage of untreated control (medium alone). Data are the mean ± SD from three individual samples in a single experiment. *P < 0.01 compared with untreated control or among groups. **(C,D)** Transcriptional expression of *Lor* **(C)** and *Krt10* **(D)** in keratinocytes in the presence of extracellular calcium was analyzed by quantitative reverse transcription polymerase chain reaction at 3 days, and expression was normalized to the *Actb* transcript. Data are the mean ± SD from three individual samples in a single experiment. *P < 0.01 compared with untreated control (extracellular calcium) or among groups. **(E)** Keratinocytes were cultured with or without huIgFc, IL-22-huIgFc, and/or IL-22BP-huIgFc in the absence of extracellular calcium for 30 min, and the expression of phosphorylated STAT3 (pSTAT3) in nucleus was analyzed by flow cytometry. Data are presented as a histogram (left panel). Data are the mean fluorescence intensity (MFI) ± SD from three individual samples in a single experiment (right panel). *P < 0.01 compared with untreated control or among groups. All data are representative of at least three independent experiments.

We further examined the impact of IL-22BP on the IL-22-mediated changes in the functionality of keratinocytes. While IL-22-huIgFc elicited the proliferation of keratinocytes in the presence or absence of extracellular calcium that triggered their differentiation, IL-22BP-huIgFc, but not huIgFc, almost completely inhibited their proliferation (Figures 4A,B). Furthermore, IL-22-huIgFc reduced the expressions of the transcripts of terminal differentiation marker genes (*Lor* and *Krt10*) of keratinocytes (Figures 4C,D). However, IL-22BP-huIgFc, but not huIgFc, inversely regulated the IL-22-mediated changes in the transcripts (Figures 4C,D). On the other hand, IL-22-huIgFc induced the translocation of phosphorylated STAT3 (pSTAT3), but not pSTAT1 and STAT5, into the nucleus in keratinocytes (37), whereas IL-22BP-huIgFc impaired the IL-22-mediated change in pSTAT3 (Figure 4E; Figure S6 in Supplementary Material).

Collectively, these results indicate that IL-22BP suppresses IL-22-mediated functional changes in keratinocytes.

### Deficiency in IL-22BP Skews the IL-17–IL-22 Cytokine Axis for Establishing Skin Inflammation

**Figure 5:**
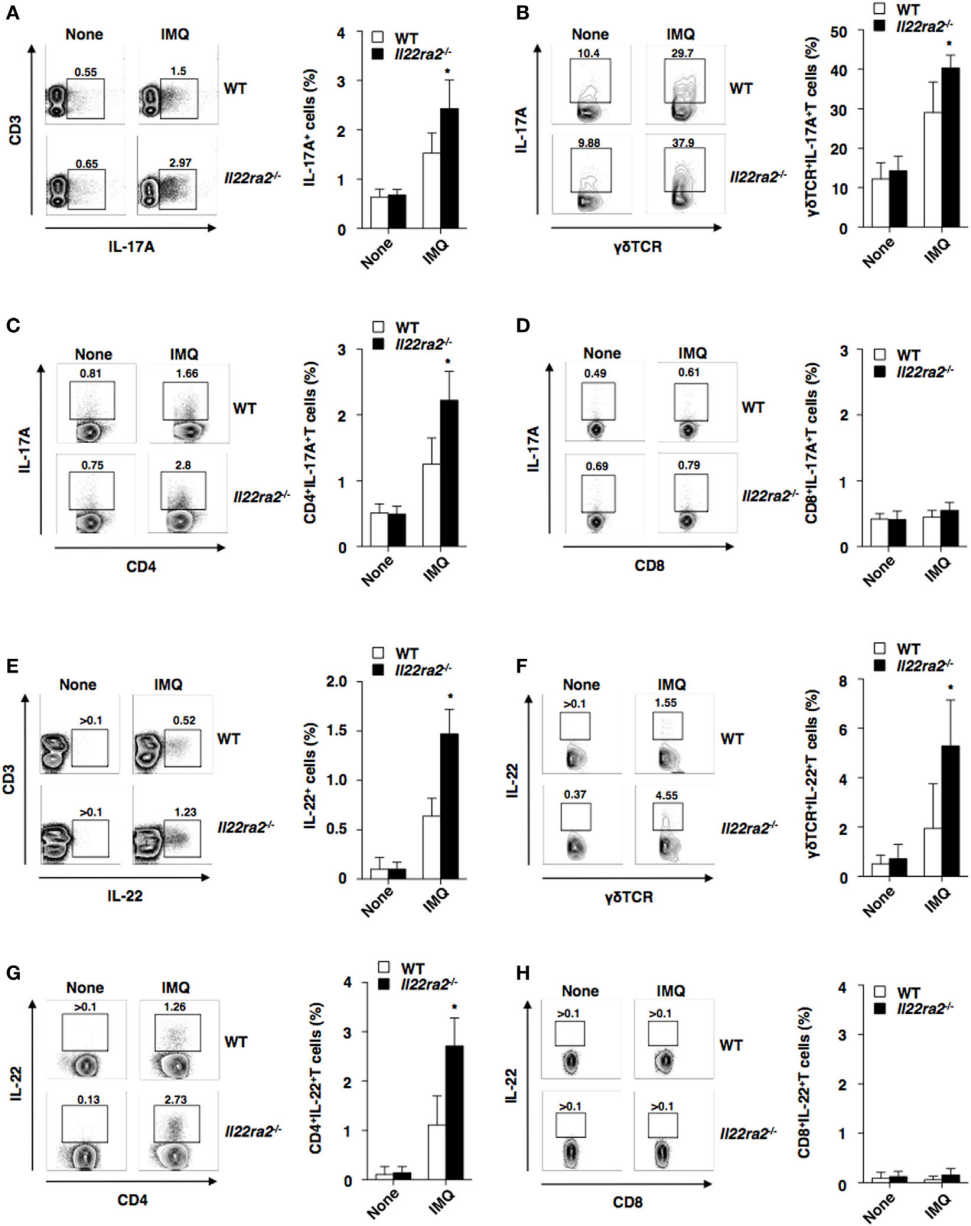
IL-22 binding protein deficiency increases the generation of lymphocytes producing interleukin (IL)-17A or IL-22 during the development of psoriatic inflammation. The frequency of IL-17A-producing cells **(A–D)** and IL-22-producing cells **(E–H)** among CD3^+^ T cells **(A,E)**, γδTCR^+^ T cells **(B,F)**, CD4^+^ T cells **(C,G)**, and CD8^+^ T cells **(D,H)** in the skin-draining peripheral lymph nodes was analyzed by flow cytometry at days 0 and 8 after topical application of imiquimod (IMQ) on the ear skin in wild-type (WT) mice (*n* = 5) and *Il22ra2^−/−^* mice (*n* = 5). Data are presented as a contour plot, and numbers represent the proportion of the indicated cell populations in each gate (left panel). Data are the mean ± SD from five individual samples in a single experiment (right panel). *P < 0.01 compared with WT mice. All data are representative of at least three independent experiments.

The above study suggests that the augmentation of IL-22 signaling is implicated to directly mediate the alternation of epithelial functionality for the progression of skin inflammatory pathogenicity in the absence of IL-22BP without affecting the function of immune cells. This is because the expression of IL-22R1 is restricted to keratinocytes in the skin. On the other hand, IL-17-producing lymphocytes have been shown to play a critical role in the development of psoriasiform dermatitis (38, 39). To verify how IL-22 signaling in epithelial keratinocytes controls the IL-17 cytokine axis to elicit the development of psoriasiform inflammation, we addressed the influence of IL-22BP deficiency on the generation of lymphocytes producing IL-17A or IL-22 in the skin-draining PLNs in psoriatic mice. Importantly, the generation of Th17 cells and IL-17A-producing γδTCR^+^ T cells, but not IL-17A-producing CD8^+^ T cells, was significantly enhanced in the absence of IL-22BP (Figures 5A–D). Similarly, IL-22BP deficiency markedly promoted the generation of Th22 cells and IL-22-producing γδTCR^+^ T cells, while it had almost no effect on the induction of IL-22-producing CD8^+^ T cells (Figures 5E–H).

It has been shown that serum amyloid A1 (SAA1) participates in the functional differentiation of Th17 cells in the intestines (34–36). As increased cutaneous expression of the *Saa1* transcript was observed in the absence of IL-22BP (Figure 2), we examined the influence of SAA1 on the generation of Th17 cells and Th22 cells *in vitro* (Figure S7 in Supplementary Material). Stimulation of CD4^+^ T cells with SAA1 enhanced the generation of Th17 cells and Th22 cells in the presence of IL-6 or IL-6 plus TGF-β.

Taken together, these results indicate that the absence of IL-22BP promotes the formation of the pathogenic IL-17–IL-22 cytokine milieu to induce psoriatic inflammation.

### IL-22BP Protects Against the Development of Psoriatic Inflammation

**Figure 6:**
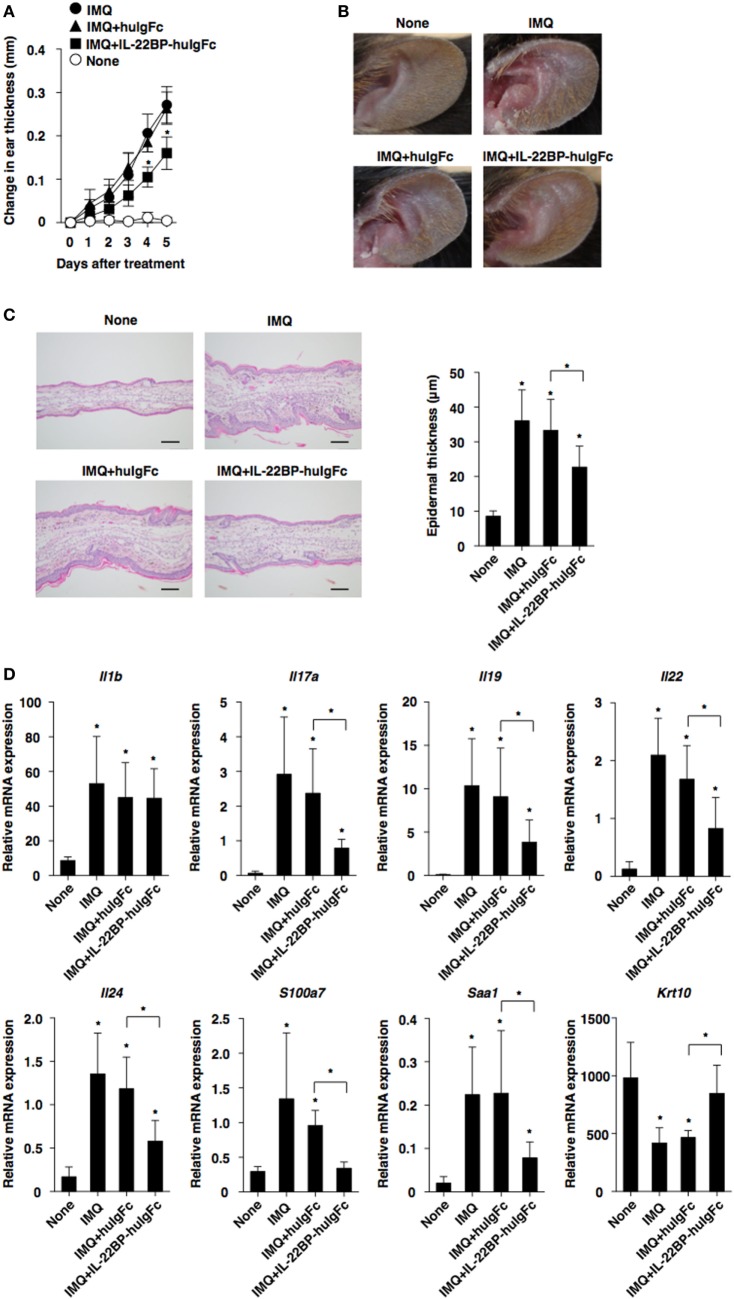
Protection of IL-22 binding protein (IL-22BP) against the development of imiquimod (IMQ)-induced psoriasiform dermatitis. Wild-type mice (each *n* = 5) that had received topical application of IMQ on the left ear skin every day for 5 days were treated with or without Fc fragment of human immunoglobulin (huIgFc) or IL-22BP-huIgFc every other day. **(A)** Ear thickness was evaluated at 5 days. Data were obtained from five individual samples in a single experiment. *P < 0.01 compared with untreated control. **(B)** Representative pictures of ear skin lesions at 5 days. **(C)** Representative hematoxylin and eosin sections (magnification; 20×) of ear skin at days 0 and 5 (left panel), and epidermal thickness was evaluated at 5 days (right panel). Data were obtained from five individual samples in a single experiment. *P < 0.01 compared with untreated control. **(D)** Transcriptional expression of cytokines and epithelial inflammation-related molecules in ear skin were analyzed by quantitative reverse transcription polymerase chain reaction at 5 days, and expression was normalized to the *Actb* transcript. Data are the mean ± SD from three individual samples in a single experiment. *P < 0.01 compared with untreated control. All data are representative of at least three independent experiments.

To further demonstrate the protective effect of IL-22BP against skin inflammation, we examined the influence of IL-22BP-huIgFc on the progression of IMQ-induced psoriatic dermatitis in WT mice. Treatment with IL-22BP-huIgFc, but not huIgFc, alleviated the severity of psoriatic pathology as confirmed by histological analyses in psoriatic mice as compared with untreated controls (Figures 6A–C). Furthermore, IL-22BP-huIgFc, but not huIgFc, attenuated the changes in the transcriptional expression levels of *Il17a, Il19, Il22, Il24, S100a7*, and *Saa1*, as well as *Krt10* and *Il25* (40) in psoriatic mice (Figure 6D; Figure S8 in Supplementary Material).

Collectively, these results indicate that treatment with IL22BP not only controls protection against inflammatory pathogenesis but also inhibits the malfunction of IL-22 signaling in keratinocytes during the development of psoriatic dermatitis.

## Discussion

Accumulating results suggest that IL-22 mediates epithelial integrity through membrane-bound IL-22R1 to control antimicrobial immunity, inflammation and tissue repair at barrier surfaces. Although dysregulated function of IL-22 is thought to be involved in the initiation and progression of epithelial pathogenesis, how epithelial IL-22 signaling is regulated in the skin remains unclear. In contrast to cDCs acting as main producers of IL-22BP to control epithelial functionality in the gastrointestinal tract, we showed the critical role of IL-22BP in mediating autoregulation of IL-22 signaling in keratinocytes to maintain skin homeostasis. Furthermore, IL-22BP protected against the development of psoriatic dermatitis mediated through abortive epidermal hyperplasia and inflammation.

Different from the intestinal mucosal tissues and secondary lymphoid tissues, in which cDCs are the major source of IL-22BP (21–23), epidermal keratinocytes and dermal mesenchymal cells exhibited its constitutive prominent production in the skin. Furthermore, the production of IL-22BP was reduced in the skin and skin-draining PLNs during the development of psoriasiform dermatitis. While stimulation with RA enhanced production of IL-22BP in cDCs (21), this stimulation somewhat diminished its production in keratinocytes. On the other hand, analysis of GF mice revealed that the steady-state production of IL-22 could require cutaneous microbial stimulation in the skin, whereas the cutaneous colonization of commensals could not affect the constitutive production of IL-22BP under the physiological condition. Collectively, these results suggest that the autocrine loop of IL-22BP constitutes a key element for regulating the responsiveness of epidermal keratinocytes to IL-22 signaling under the steady-state condition, whereas inflammatory stimulation abrogates their production of IL-22BP under the pathological condition. Although the precise mechanism responsible for the initiation of IL-22BP production in keratinocytes remains unclear, its transcript may be upregulated during the differentiation of keratinocytes from their progenitors. Further study will be needed to test this possibility.

While the pathogenesis of psoriasis involves several axes of immune dysregulation, the impact of IL-22BP on the epithelial functionality leading to the development of this cutaneous immune pathology remains unclear. Our results clearly showed that IL-22BP deficiency aggravated the incidence and severity of psoriatic dermatitis, accompanied by massive acanthosis and parakeratosis, as well as dermal leukocytic infiltration. Indeed, the absence of IL-22BP exacerbated IL-22-induced psoriatic dermatitis. Furthermore, IL-22BP deficiency led to enhanced or reduced expressions of AMPs (*Reg3g* and *S100a7*) and acute phase protein (*Saa1*) or terminal differentiation marker genes (*Lor* and *Krt10*) in keratinocytes. Taken together, these results suggest that IL-22BP works with epithelial autoregulation of IL-22 signaling in keratinocytes to control epithelial functionality to avoid undesirable skin inflammation.

Despite the restricted expression of IL-22R1 on non-hematopoietic cells allowing them to respond to IL-22 (12–14), IL-22BP deficiency caused drastic enhancement of cutaneous production of various inflammatory cytokines and chemokines, as well as the infiltration and accumulation of inflammatory leukocytes in the skin and skin-draining PLNs in psoriatic mice. Furthermore, the augmented transcriptional expression of several cytokines (*Il19, Il20*, and *Il24*) as sources of keratinocytes (5, 41) was observed in the pathological sites in the absence of IL-22BP. As the IL-20 subfamily of these cytokines secreted by keratinocytes has been implicated not only to act on various types of immune cells and non-hematopoietic cells but also to cause their further activation through a positive feedback loop, our finding suggests that IL-22BP impair the IL-22-mediated activation of keratinocytes to elicit the IL-19/IL-20/IL-24 axis, and this results in the amelioration of psoriatic dermatitis.

Although it has been shown that IL-22BP blocks the binding of IL-22 to IL-22R1 (18–20), how IL-22BP regulates the IL-22-mediated functional changes in keratinocytes remains unknown. We showed that IL-22BP almost completely inhibited the increased proliferation of keratinocytes and their aberrant differentiation upon stimulation with IL-22. Furthermore, IL-22BP impaired their IL-22-induced nuclear translocation of pSTAT3. Thus, IL-22BP could abrogate the IL-22-mediated alternation of keratinocyte functionality to alleviate acanthosis and parakeratosis, as well as skin inflammation during the progression of psoriatic dermatitis.

Growing evidence has shown that IL-23–IL-17 cytokine axis is a critical in the pathogenesis of psoriasis development (38, 39). While IL-23 produced by macrophages and cDCs reportedly participated in IL-17 secretion from Th17 cells and γδT cells as well as ILC3 (38, 39), IL-22BP deficiency had no effect on the expression of *Il23a* in psoriatic skin, implying that the IL-22BP-mediated impairment of IL-22 signaling does not contribute to control the ability of these phagocytes to produce IL-23. On the other hand, we showed that the generation of Th17 cells and IL-17A-producing γδTCR^+^ T cells, as well as Th22 cells and IL-22-producing γδTCR^+^ T cells were markedly enhanced in skin-draining PLNs under deficiency of IL-22BP. Previous studies have shown that IL-1β and SAA1 potentiated the production of IL-17A and IL-22 in Th17 cells and Th22 cells (34–36) as well as γδTCR^+^ T cells (42, 43) in mucosal tissues. Indeed, the absence of IL-22BP resulted in the enhanced transcriptional expression of *Il1b* and *Saa1* in psoriatic skin, and stimulation with SAA1 enhanced the generation of Th17 cells and Th22 cells under Th17- and Th22-polarized culture conditions. Collectively, these results suggest that IL-22BP inhibits the formation of the IL-1β-SAA1-enriched milieu, which accelerates the generation of IL-17/IL-22-producing lymphocytes and their activation, mediated through the abortive IL-22-mediated activation of keratinocytes.

In accordance with the potent inhibitory function of IL-22BP on the IL-22-mediated functional alternation of keratinocytes *in vitro*, treatment of psoriatic mice with IL-22BP exerted marked protection against the development of skin pathogenesis, possibly due to the blockade of abnormal changes of keratinocytes. As the observed function of IL-22BP to alleviate psoriatic disease onset is comparable to that of anti-IL-22 monoclonal Ab (14), the use of IL-22BP could be a candidate treatment of this pathogenesis. Taken together, these results suggest that IL-22BP neutralizes the pathogenic actions of IL-22 signaling in keratinocytes to ameliorate psoriatic dermatitis.

In conclusion, we described the critical role of IL-22BP in mediating autoregulation of IL-22 signaling in keratinocytes to control cutaneous pathogenesis, and therefore fine tuning of the balance in the IL-22–IL-22R1–IL-22BP axis is essential to maintain epithelial homeostasis to evoke antimicrobial immunity, tissue repair at barrier surfaces, and prevent undesirable skin inflammatory disorders. Thus, IL-22BP may constitute an attractive target for the intervention and treatment of skin autoimmune and inflammatory disorders.

## Ethics Statement

This study was carried out in accordance with the recommendations of Japanese national guidelines and institutional review board of University of Miyazaki. The protocol was approved by the Institutional Animal Care and Use Committee of University of Miyazaki.

## Author Contributions

KS designed all experiments, analyzed data, and wrote the manuscript. TFukaya, TFukui, TU, HT, JN, NM, KA, TN, and NC did experiments. HK and YH provided reagents and information.

## Conflict of Interest Statement

The authors declare that the research was conducted in the absence of any commercial or financial relationships that could be construed as a potential conflict of interest.

## Abbreviations

Ab, antibody; BM, bone marrow; BM-cDCs, BM-derived conventional dendritic cells; DT, diphtheria toxin; DTR, DT receptor; EGFP, enhanced green fluorescent protein; FAE, follicle-associated epithelium; GF, germ-free; H&E, hematoxylin and eosin; huIgFc, Fc fragment of human immunoglobulin; IBD, inflammatory bowel diseases; i.d., intradermal; IFN, interferon; IFN-I, type I IFN; IgG, immunoglobulin G; IL, interleukin; ILCs, innate lymphoid cells; IL-10R, IL-10 receptor; IL-22BP, IL-22 binding protein; IL-22R, IL-22 receptor; IMQ, imiquimod; i.p., intraperitoneally; mAb, monoclonal Ab; MFI, mean fluorescence intensity; MLNs, mesenteric lymph nodes; PCNA, proliferating cell nuclear antigen; pDCs, plasmacytoid DCs; PLNs, peripheral lymph nodes; PP, Payer’s patch; pSTAT, phosphorylated signal transducer and activator of transcription; RA, retinoic acid; RT-qPCR, quantitative reverse transcription polymerase chain reaction; SAA, serum amyloid A; SPF, specific pathogen-free; Spleen, Spl; Teff, effector T; TGF, transforming growth factor; Th, T helper; TLR, toll-like receptors; TNF, tumor necrosis factor; WT, wild type.
