# Supplementary material for: Pivotal Role of IL-22 Binding Protein in the Epithelial Autoregulation of Interleukin-22 Signaling in the Control of Skin Inflammation
Source: Front Immunol. 2018 Jun 21;9:1418. doi: 10.3389/fimmu.2018.01418 (PMC6021537; doi:10.3389/fimmu.2018.01418)
Supplement: Supplementary file 1 [file Presentation_1.PDF]

Supplementary Material

Pivotal Role of IL-22BP in the Epithelial Autoregulation of IL-22 Signaling in the Control of Skin Inflammation

Tomohiro Fukaya, Takehito Fukui, Tomofumi Uto, Hideaki Takagi, Junta Nasu, Noriaki Miyanaga, Keiichi Arimura, Takeshi Nakamura, Haruhiko Koseki, Narantsog Choijookhuu, Yoshitaka Hishikawa, and Katsuaki Sato*

***Correspondence:** Katsuaki Sato: katsuaki_sato@med.miyazaki-u.ac.jp

SUPPLEMENTARY MATERIALS AND METHODS

Generation of *Il22ra2*$^{-/-}$ Mice

The targeting vector for *Il22ra2*$^{-/-}$ mice was constructed in the pBluescript vector by using a 2.0-kilobase (kb) genomic fragment (left arm) upstream of *Il22ra2* exon 1, and a 4.0-kb genomic fragment (right arm) downstream of exon 1 cloned from a modified bacterial artificial chromosome (BAC) clone, RP23-353K11 (Children's Hospital Oakland Research Institute), containing the complete *Il22ra2* gene. The left and right arm were custom-made using GeneArt® (Life Technologies), and each of the 5'- and 3'-ends was tagged with *Xho*I and *Sal*I sites for the left arm or *Sal*I and *Cla*I sites for the right arm, respectively. Following the digestion of the 2.0-kb fragment with *Xho*I and *Sal*I, and the 4.0-kb PCR fragment with *Sal*I and *Cla*I, each fragment was ligated into each site of pBluescript. A *Sal*I restriction site was engineered in place of the start codon in exon 1. The *pStop-loxP-Cre/Neo*^{r}*-loxP* auto-deleter cassette (44-46) was cloned into the *SalI* site inserted into the targeting vector. Finally, the targeting construct was abutted to a PMC1-DTa negative-selection cassette and linearized. The linearized targeting construct was introduced by electroporation into C57BL/6-derived JN/2 recombinant embryonic stem cell (ESC) and neomycin-resistant clones were first screened for homologous recombination by PCR utilizing a pair of the following oligonucleotides: Primer 1 (5'-GCT TCA AAG GTA GAT TCA TCA GTG T-3') and Primer 4 (5'-ATA TAG ACG TTG TGG CTG TTG TAG TTG TA-3'). *Kpn*I-digested genomic DNA of positive clones was then screened by Southern blotting with a 3' external single-copy probe corresponding to a 0.599-kb fragment, which was amplified by PCR using these primers: 5'-TCA AAC CGT TAA CTC CAA TAA ACT C-3' and 5'-TGG ATC ACA CTT TGT TGA ATA TTT G-3'. When tested on *Kpn*I-digested DNA, it hybridized either to a 13.9-kb WT fragment or to a 10.9-kb recombinant fragment. ESC clones bearing the correctly targeted locus were injected into BALB/c blastocysts, and chimeric male offspring, in which the auto-deleter cassette was self-excised during the male germline transmission, were mated with female C57BL/6 mice to obtain heterozygotes, which were then crossed to obtain homozygotes. Transmission of the targeted allele was confirmed by PCR with Primer 2 (5'-GTT GTT GAA GAT AAT TTC TCA ATC T-3') and Primer 3 (5'-GTT GTC AAA TAA TAA AAG TGA GTT CCT G-3'). The mutant mice were cross-mated for more than nine generations with C57BL/6 mice, and 8- to 12-week-old *Il22ra2*$^{+/+}$ littermates were used as WT mice.

Tissue and Cell Isolation

To prepare single-cell suspensions from Spl, PLNs, MLNs, thymus, liver, lung, kidney, and PP (44-46), tissues were digested with 400 U/ml collagenase type III (Worthington Biochemical) at 37 ^{o}C for 40 or 60 min, and were ground between glass slides. Cell suspensions of Spl, lung, and kidney were further treated with RBC lysis buffer (Sigma-Aldrich). BM cells were flushed from the femurs and tibias. Single-cell suspensions were obtained by forcing through a 40-μm cell strainer (BD Biosciences). Back skin fragments were dissected. To isolate intestinal leukocytes (45), the small intestine and colon were opened longitudinally, washed to remove fecal content, and cut into small pieces. To remove epithelial cells, intestinal segments were treated in PBS containing 10 % FCS, 20 mM HEPES, 100 U/ml penicillin, 100 μg/ml streptomycin, 1mM sodium pyruvate, 20 mM EDTA and 10 μg/ml polymyxin B (Calbiochem) with continuous stirring at 37 ^{o}C for 20 min in a water bath. After washing with PBS, the remaining tissues were incubated with 400 U/ml collagenase type III, 250 mU/ml dispase (BD Biosciences), and 100 μg/ml DNase I (Roche Diagnostics) for 20-30 min at 37 ^{o}C in a water bath. The cell suspension was prepared by forcing through a 100-μm cell strainer, washing with PBS, resuspended in 10 ml of 30% Percoll (GE Healthcare) and overlaying on 2 ml of 70% percoll in a 15-ml tube. Percoll gradient separation was performed by centrifugation at 780 *g* for 20 min at room temperature. The LP leukocytes were collected at the interface of the Percoll gradient and washed with RPMI1640/10% FCS, and used immediately for experiments. CD11c^{+} DCs and BST2^{+} pDCs were purified by AutoMACS with mouse CD11c (N418) Microbeads and a mouse pDC isolation kit II (both from Miltenyi Biotec). Subsequently, CD11c^{+} DCs or BST2^{+} pDCs were sorted into CD11c^{high}B220^{-}BST2^{-} cDCs or CD11c^{int}B220^{+}BST2^{+} pDCs with high purity (each >99%) using a FACSAriaII cell sorter with fluorescein-conjugated mAbs (BD Biosciences). B220^{+} B cells, CD3^{+}CD4^{+} T cells, CD3^{+}CD8^{+} T cells were purified from splenocytes using a FACSAriaII cell sorter with fluorescein-conjugated mAbs (BD Biosciences). To obtain a single-cell suspension from ears, ear halves were separated and cut into small pieces. Ear segments were digested with 400 U/ml collagenase type III at 37 ^{o}C for 50 min, and the tissues were mechanically disrupted and pressed through a 100-μm cell strainer. To prepare epidermal and dermal skin-resident cells, ear halves were separated and incubated in 0.1% Trypsin in PBS at 37 ^{o}C for 50 min. Subsequently, dermis and epidermis were separated, cut into small pieces, and digested in PBS containing 0.1% Trypsin and 0.1 mg/ml DNase at 37 ^{o}C for 30 min. The dermis was further digested with 400 U/ml collagenase type III at 37 ^{o}C for 30 min. Single-cell suspensions were separated into CD45^{-} epidermal keratinocytes, CD45^{-} dermal mesenchymal cells, CD45^{+}I-A/I-E^{-} leukocytes, and CD45^{+}I-A/I-E^{+} leukocytes by a FACSAriaII cell sorter with fluorescein-conjugated mAbs (BD Biosciences).

Flow Cytometry

Cells were stained with fluorescein-conjugated mAbs and Ab to mouse, CD4 (RM4-5), CD8a (53-6.7), CD11c (HL3), CD45R/B220 (RA3-6B2), CD45.2 (104), I-A/I-E (M5/114.15.2), Gr-1 (RB6-8C5), IL-17A (TC11-18H10), isotype-matched control mAb (BD Biosciences), CD3ε (145-2C11), CD11b (M1/70), CD44 (1M7), CD62L (MEL-14), γδTCR (GL3), IL-22 (IL22JOP) (eBiosciences), and mPDCA-1/BST2 (JF05-1C2.4.1) (Miltenyi Biotec). For the intracellular expression of cytokines, cells were incubated for 4 hrs with phorbol 12-myristate 13-acetate (PMA, 50 ng/ml; Sigma-Aldrich) and ionomycin (500 ng/ml; Sigma-Aldrich) plus GolgiPlug (BD Biosciences) during the final 2 hrs. Subsequently, the cells were resuspended in Fixation-Permeabilization solution (eBiosciences) and intracellular cytokine staining was carried out according to the manufacturer's directions.

Fluorescence staining was analyzed with a FACSVerse flow cytometer (BD Biosciences) and FlowJo software (Tree star).

Quantitative RT-PCR

Total RNA from colonic explants was extracted with Trizol (Life Tecnologies) and the first-strand complementary DNA (cDNA) was synthesized from 1 μg of total RNA using the PrimeScript RT Master Mix (Takara) according to the manufacturer's instructions. Transcriptional expression levels were analyzed by using SYBR® Premix Ex Taq II on Thermal Cycler Dice (Takara) with specific primer pairs (**TABLE S1**) after normalization for the expression of *Actb* or *Gapdh* (46).

Generation of Human IgG Fc Fusion Protein

DNA sequences containing the extracellular domain lacking the signal sequence of *Il22* (NCBI accession No. NM_016971, amino acids [aa]: 34-179) and *Il22ra2* (NCBI accession No. NM_178258, amino acids [aa]: 21-230) were custom-made using GeneArt®, and the 5'- and 3'-ends of the synthesized DNA sequences were also tagged with *EcoR*I and *Bgl*II sites, respectively. After restriction enzyme digestion, the synthesized DNA sequence was cloned into the sites of pFUSE-hIgG2-Fc2 (Invivogen), and transfected into FreeStyle™ 293-F cells (Life Technologies) using 293fectin™ Transfection Reagent (Life Technologies) according to the manufacturers' instructions (46). huIgFc, IL-22-huIgFc, or IL-22BP-huIgFc fusion protein was purified from the culture supernatant using HiTrap™ Protein G HP (GE Healthcare Life Sciences), and the purified fusion protein was separated by 10% SDS-PAGE under non-reducing or reducing conditions, respectively, and stained with a CBB Stain kit (Bio-Rad Laboratories) to confirm the protein products.

In vitro CD4$^+$ T-cell Differentiation Assay

To differentiate Th17 cells (30, 46), naïve CD4$^+$ T cells (4x10^4) were cultured with cDCs (2x10^4) in the presence or absence of SAA1 (5 μg/ml; R&D Systems) in combination with anti-CD3ε mAb (5 μg/ml; 145-2C11, Biolegend), anti-IFN-γ mAb (10 μg/ml; R4-6A2, BD Biosciences), anti-IL-4 mAb (10 μg/ml; 11B11, BD Biosciences), recombinant human TGF-β1 (2 ng/ml; Wako Pure Chemicals), and recombinant mouse IL-6 (40 ng/ml; Wako Pure Chemicals) for 3 days in 96-well round-bottomed plates (BD Bioscience). To differentiate Th22 cells, CD4$^+$ T cells (4x10^4) were cultured with cDCs (2x10^4) in the presence or absence of SAA1 (5 μg/ml) in combination with anti-CD3ε mAb (5 μg/ml; 145-2C11), anti-IFN-γ mAb (10 μg/ml; R4-6A2), anti-IL-4 mAb (10 μg/ml; 11B11), and recombinant mouse IL-6 (40 ng/ml) for 3 days in 96-well round-bottomed plates (BD Bioscience). Analysis of IL-17A or IL-22 expression among gated CD4$^+$ T cells was performed by flow cytometry as described above.

Cell Culture

Murine keratinocytes (COCA, ECACC) were stimulated with or without huIhFc, IL-22-huIgFc, and/or IL-22BP-huIgFc (each 5 μg/ml) in CnT-07 medium (CELLnTEC) in the presence or absence of extracellular calcium (1.2 mM) for 3 days. cDCs were prepared by culturing BM cells with murine granulocyte-macrophage colony-stimulating factor (GM-CSF, 20 ng/ml, Wako Pure Chemical Industries) as described elsewhere in the presence or absence of RA (100 nM; Sigma-Aldrich) for 8 days. Subsequently, cells were collected and used for RNA preparation. In some experiments, proliferation of keratinocytes was determined by using the Premix WST-1 cell proliferation assay system (Takara) according to the manufacturer's instructions. Analysis of the intranuclear expression of pSTAT3 in keratinocytes was performed according to previous reports (10, 37) with some modification. In brief, keratinocytes were cultured with or without huIgFc, IL-22-huIgFc, and/or IL-22BP-huIgFc (each 5 μg/ml) in CnT-07 medium (CELLnTEC) in the absence of extracellular calcium for 30 min, and cells were resuspended in Lise/Fix Buffer for 10 min at 37 ^{o}C (BD Biosciences) followed by permeabilization in Perm Buffer III for 30 min on ice (BD Biosciences). Subsequently, intranulear staining with Alexa Fluor647 Mouse Anti-Stat1 (pY701, 4a; BD Biosciences), Alexa Fluor647-conjugated anti-pSTAT3 mAb (pY705, 4/P-STAT3; BD Biosciences), and PE Mouse Anti-Stat5 (pY694, 47/Stat5; BD Biosciences) was carried out according to the manufacturer's directions with flow cytometry as described above.

Skin Inflammation

A mouse model of IMQ-induced psoriasiform skin inflammation was established as previously (30). In brief, mice were treated topically with either 25 mg of 5% IMQ cream (Mochida Pharmaceutical) or petrolatum (Wako Pure Chemicals) as a control on the left ear every day for 8 days. To detect the effect of IL-22 or IL-22BP on the development of psoriatic dermatitis, mice were injected i.d. in the left ear with or without huIgFc, IL-22-huIgFc and/or IL-22BP-huIgFc (each 10 μg/mouse) every day and/or every other day for 4 or 5 days. The severity of the ear skin inflammation of each mouse was determined daily by ear thickness using digital calipers (PK-1012CPX; Mitsutoyo), and ear photographs were taken at days 0 and 5 or 8 after topical application of IMQ cream on the ear skin. In some experiments, the ear skin of each mouse was obtained daily after topical application of IMQ cream on the ear skin for 5 or 8 days. Similarly, the ear skin was collected at days 8 after topical application of RA (40 nmol/20 μl in ethanol).

Histopathological Assessment

Ear tissues were fixed with 4% paraformaldehyde (PFA) in PBS and embedded in paraffin. The tissue sections (5 μm thickness) were stained with H&E. The stained slides were examined with a bright-field microscope (BX53; Olympus). The thickness of ear epidermis and dermis was measured for thickness using ImageJ (National Institutes of Health) by a blinded observer as described previously (30).

Immunohistochemical Analysis

To detect PCNA (30), 4% PFA-fixed paraffin-embedded sections of the ear skin (5 μm thickness) were deparaffinized with toluene and rehydrated through a graded ethanol series. After inactivation of endogenous peroxidase with 0.3% H_2O_2 in methanol for 15 min and blocking with normal goat IgG, the sections were reacted overnight with the primary Ab against PCNA (PC10, Dako) in 1% BSA in PBS. After reaction with an HRP-conjugated goat anti-mouse IgG F(ab)' second Ab, the sites of HRP were visualized with DAB and H_2O_2. The stained slides were analyzed as described above. For cutaneous detection of Gr-1$^+$ granulocytes and γδTCR$^+$ T cells (30), the ear skin was embedded in OCT compound (Sakura Fineteck) and frozen in liquid N_2. The tissue block was sectioned with a cryostat at 5-7 μm. Frozen sections were fixed with cold acetone and blocked in PBS containing 5% normal rat serum. Subsequently, slide was stained with FITC-conjugated anti-Gr-1 mAb (BD Biosciences) or APC-conjugated anti-gdTCR (Biolegend) mAb and mounted with Vectashield (Vector laboratories). The stained slides were analyzed with a All-in-One Fluorescence Microscope (BZX-710; KEYENCE).

SUPPLEMENTARY REFERENCES

44. Takagi H, Fukaya T, Eizumi K, Sato Y, Sato K, Shibazaki A, et al. Plasmacytoid dendritic cells are crucial for the initiation of inflammation and T cell immunity in vivo. Immunity (2011) 35(6):958-71. doi:10.1016/j.immuni.2011.10.014
45. Fukaya T, Murakami R, Takagi H, Sato K, Sato Y, Otsuka H, et al. Conditional ablation of CD205$^+$ conventional dendritic cells impacts the regulation of T-cell immunity and homeostasis in vivo. Proc Natl Acad Sci U S A (2012) 109(28):11288-93. doi:10.1073/pnas.1202208109
46. Uto T, Fukaya T, Takagi H, Arimura K, Nakamura T, Kojima N, et al. Clec4A4 is a regulatory receptor for dendritic cells that impairs inflammation and T-cell immunity. Nat Commun (2016) 7:11273. doi:10.1038/ncomms11273
47. Arimura K, Takagi H, Uto T, Fukaya T,Nakamura T, Choijookhuu N, et al. Crucial role of plasmacytoid dendritic cells in the development of acute colitis through the regulation of intestinal inflammation. Mucosal Immunol (2017) 10(4):957-70. doi:10.1038/mi.2016.96

Table S1

RT-PCR primer	Sequence
Actb	F-5'-ATCATTGCTCCTCCTGAGCG-3'
	R-5'-GCTGATCCACATCTGCTGGAA-3'
Gapdh	F-5'-AAATTCAACGGCACAGTCAAG-3'
	R-5'-TGGTGGTGAAGACACCAGTAG-3'
Il1b	F-5'-GAAGAAGAGCCCATCCTCTG-3'
	R-5'-TCATCTCGGAGCCTGTAGTG-3'
Il6	F-5'-GACTGATGCTGGTGACAACC-3'
	R-5'-CCTCCGACTTGTGAAGTGG-3'
Il17a	F-5'-CTGCTGAGCCTGGCGGCTAC-3'
	R-5'-CATTGCGGTGGAGAGTCCAGGG-3'
Il19	F-5'-GCCAACTCTTTCCTCTGCGT-3'
	R-5'-GGTGGCTTCCTGACTGCAGT-3'
Il20	F-5'-GACCCCTGACCACCATACCC-3'
	R-5'-CCATTGCTTCTTCCCCACAA-3'
Il22	F-5'-CAGCTCCTGTCACATCAGCGGT-3'
	R-5'-AGGTCCAGTTCCCCAATCGCCT-3'
Il22ra2	F-5'-AGTCCATACAGGAACCTTGACTTGA-3'
	R-5'-TTGAGCCTTAGGCCACCTGA-3'
Il23a	F-5'-TCCGTTCCAAGATCCTTCG-3'
	R-5'-GAACCTGGGCATCCTTAAGC-3'
Il24	F-5'-GCCCAGTAAGGACAATTCCA-3'
	R-5'-ATTTCTGCATCCAGGTCAGG-3'
Il25	F-5'-GTGGAGCTCTGCATCTGTGT-3'
	R-5'-CCCGATTCAAGTCCCTGTCC-3'
Il36a	F-5'-TGCCCACTCATTCTGACCCA-3'
	R-5'-GTGCCACAGAGCAATGTGTC-3'
Ccl20	F-5'-AATCTGTGTGCGCTGATCCA-3'
	R-5'-CCTTGGGCTGTGTCCAATTC-3'
Cxcl1	F-5'-GCCTATCGCCAATGAGCTG-3'
	R-5'-ATTCTTGAGTGTGGCTATGA-3'
Csf3	F-5'-TGCACTATGGTCAGGACGAG-3'
	R-5'-GGGGTGACACAGCTTGTAGG-3'
Mmp9	F-5'-CATTCGCGTGGATAAGGAGT-3'
	R-5'-TCACACGCCAGAAGAATTTG-3'
Reg3g	F-5'-TTCCTGTCCTCCATGATCAAAA-3'
	R-5'-CATCCACCTCTGTTGGGTTCA-3'
S100a7	F-5'-GGGCAGCTGACAAAAACAAG-3'
	R-5'-TGGAACTGGAGATGGTAGTCC-3'
Saa1	F-5'-TGTTCACGAGGCTTTCCAAG-3'
	R-5'-CCCGAGCATGGAAGTATTTG-3'
Lor	F-5'-CACATCAGCATCACCTCCTTCC-3'
	R-5'-CCTCCTCCACCAGAGGTCTTTC-3'
Krt10	F-5'-CGTACTGTTCAGGGTCTGGAG-3'
	R-5'-GCTTCCAGCGATTGTTTCA-3'

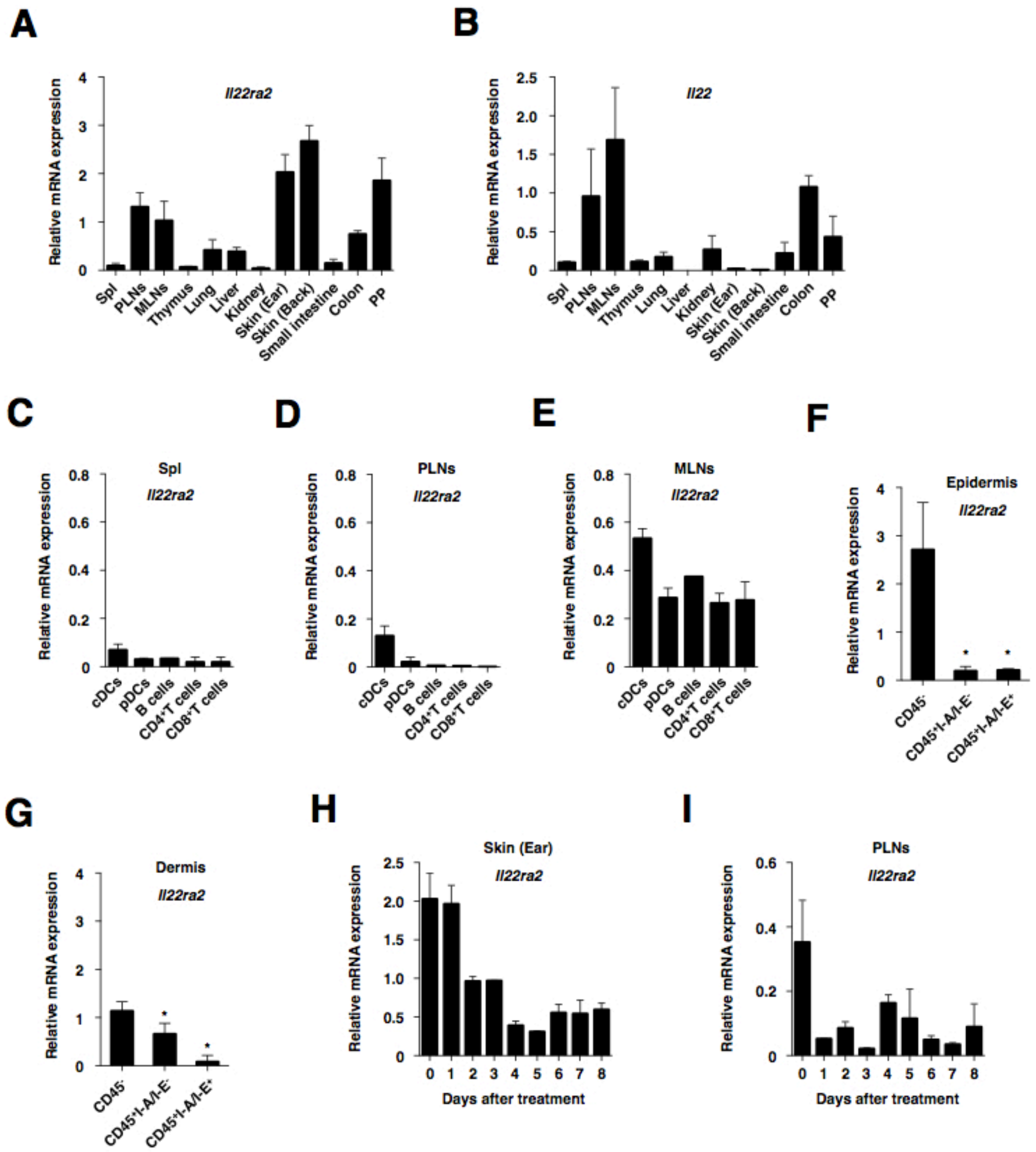

FIGURE S1. Transcriptional expression of *Il22ra2* and *Il22* in normal mice. **(A,B)** Transcriptional expression of *Il22ra2* and *Il22* in different tissues was analyzed by quantitative reverse transcriptase-PCR (RT-qPCR), and expression was normalized to the *Actb* transcript. Data are the mean ± s.d. from three individual samples in a single experiment. **(C-E)** Transcriptional expression of *Il22ra2* was compared by RT-qPCR for leukocytes in Spl **(C)**, PLNs **(D)**, and MLNs **(E)**, and expression was normalized to the *Actb* transcript. Data are the mean ± s.d. from three individual samples in a single experiment. **(F,G)** Transcriptional expression of *Il22ra2* was compared by RT-qPCR for $CD45^-$ keratinocytes **(F)**, $CD45^-$ mesenchymal cells **(G)**, $CD45^+$I-A/I-E^- leukocytes **(F,G)**, and $CD45^+$I-A/I-E^+ leukocytes **(F,G)** in the epidermis **(F)** and dermis **(G)**, and expression was normalized to the *Actb* transcript. Data are the mean ± s.d. from three individual samples in a single experiment. **P* < .01 compared with $CD45^-$ keratinocytes. **(H,I)** Transcriptional expression of *Il22ra2* in ear skin **(H)** and PLNs **(I)** was analyzed by RT-qPCR before and after topical application of IMQ on the ear skin for 8 days, and expression was normalized to the *Gapdh* transcript. Data are the mean ± s.d. from three

individual samples in a single experiment. All data are representative of at least three independent experiments.

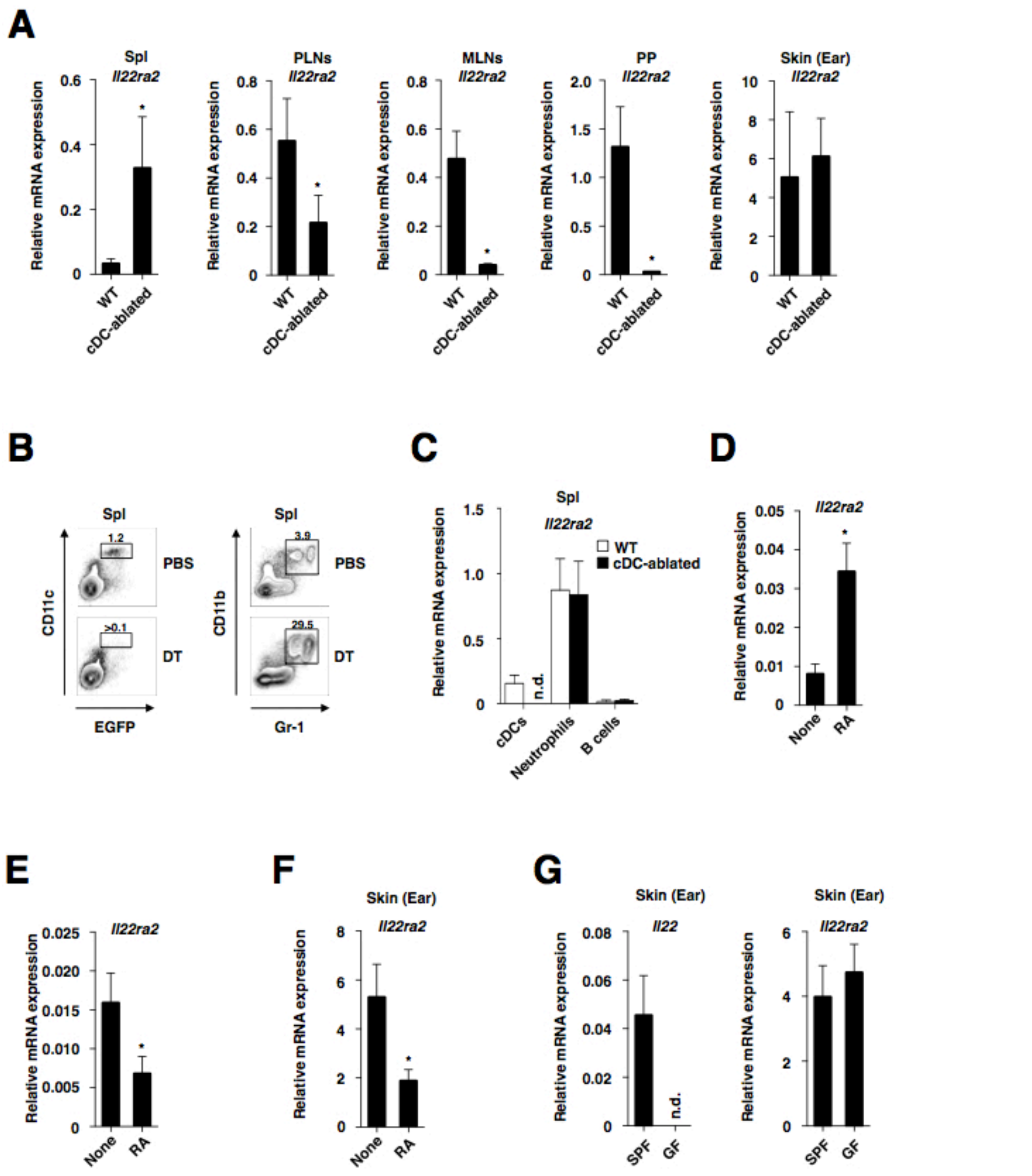

FIGURE S2. Transcriptional expression of *Il22ra2* in the absence of cDCs. **(A)** Transcriptional expression of *Il22ra2* in different tissues in WT mice and cDC-ablated mice was analyzed by RT-qPCR at 1 day after DT treatment, and expression was normalized to the *Actb* transcript. Data are the mean ± s.d. from three individual samples in a single experiment. **(B)** The frequency of $CD11c^{high}EGFP^{+}$ cDCs (left panel) and $CD11b^{+}Gr\text{-}1^{+}$ neutrophils (right panel) in Spl in cDC-ablated mice was analyzed by flow cytometry at 0 and 1 day after DT treatment. Data are presented as a contour plot, and numbers represent the proportion of the indicated cell populations in each gate. **(C)** Transcriptional expression of *Il22ra2* was compared by RT-qPCR for leukocytes in Spl in WT mice and cDC-ablated mice were analyzed by RT-qPCR at day 1 after DT treatment. Expression was normalized to the *Actb* transcript. Data are the mean ± s.d. from three individual samples in a single experiment. **(D,E)** BM-cDCs **(D)** or keratinocytes **(E)** were stimulated with or without RA for 3 days.

Subsequently, transcriptional expression of *Il22ra2* was analyzed by RT-qPCR, and expression was normalized to the *Actb* transcript. Data are the mean ± s.d. from three individual samples in a single experiment. **(F)** Transcriptional expression of *Il22ra2* in ear skin was analyzed by RT-qPCR before and after topical application of RA at 8 days on the ear skin, and expression was normalized to the *Actb* transcript. Data are the mean ± s.d. from three individual samples in a single experiment. **(G)** Transcriptional expression of *Il22* (left panel) and *Il22ra2* (right panel) in ear skin in SPF or GF WT mice was analyzed by RT-qPCR, and expression was normalized to the *Actb* transcript. Data are the mean ± s.d. from three individual samples in a single experiment. All data are representative of at least three independent experiments.

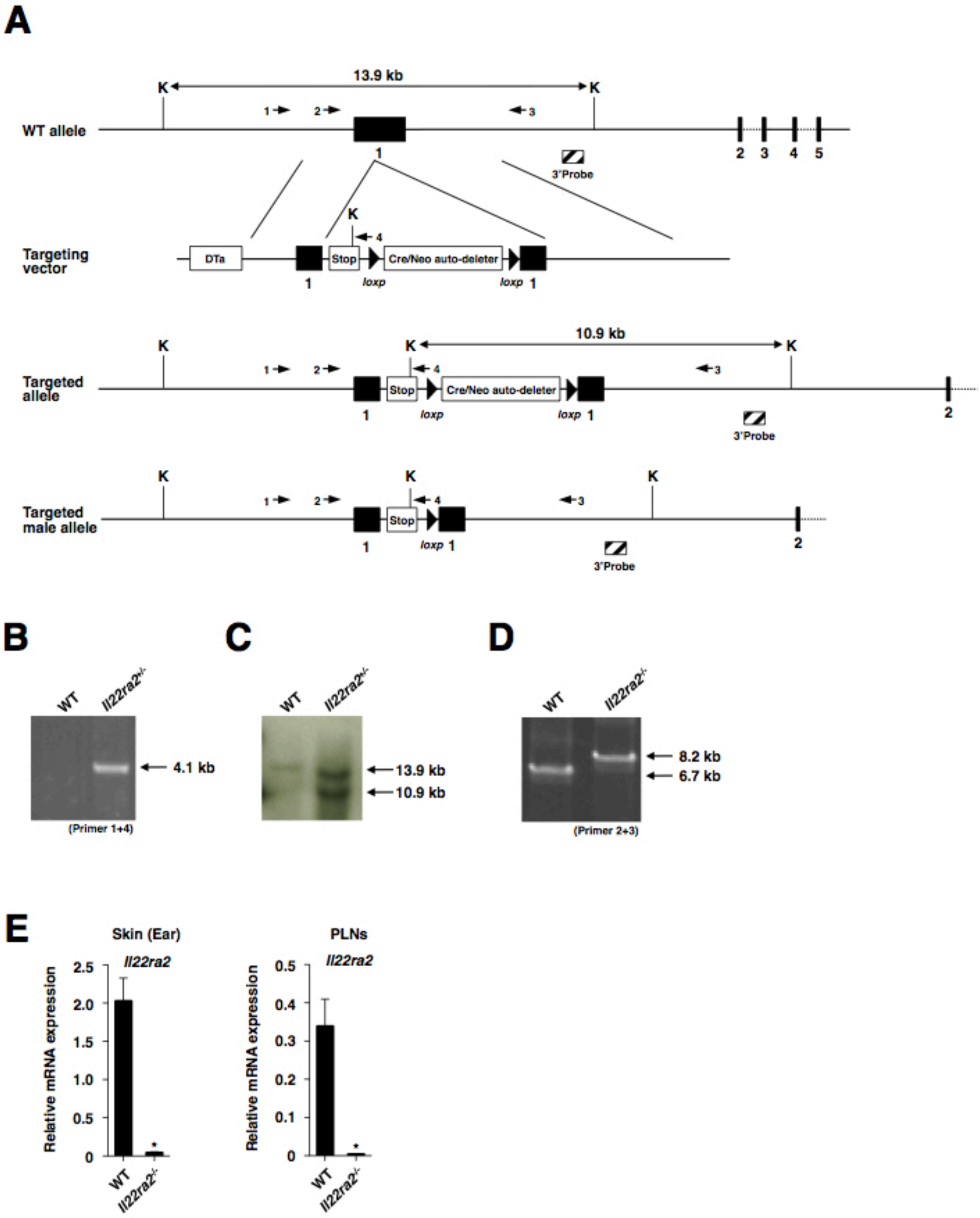

FIGURE S3. Generation and identification of *Il22ra2*$^{-/-}$ mice. **(A)** Strategy used to produce the *Il22ra2*$^{-/-}$ mice. (1) Partial restriction map of the WT *Il22ra2* gene. Exons are depicted as black boxes. The restriction site indicated is K: *Kpn*I (2) Targeting vector used to introduce the mutations in the *Il22ra2* gene. A *Sal*I site engineered in place of the start codon in exon 1 of the *Il22ra2* gene was used to clone the Stop-Cre/Neo^r auto-deleter cassette. DTa: diphtheria toxin a expression cassette, Stop: Stop cassette, Cre/Neo auto-deleter: Cre/Neo^r auto-deleter cassette. The Cre/Neo^r auto-deleter cassette is shown bracketed by Lox P sites (filled triangles); it directs its own excision as it passes through the male germline. (3) Structure of the targeted allele following homologous recombination in ESC clones. (4) Structure of the *Il22ra2* allele following expression of Cre recombinase and

excision of the Neo^r cassette in mutant mice. The 3' external single-copy probe (a hatched box) and the PCR primers at the 5' end (blackarrows) used to verify proper homologous recombination events are shown. **(B,C)** DNA-PCR **(B)** and Southern blot **(C)** analysis of WT and recombinant ESC clones. **(D)** Genotyping of tail DNA from WT mice and homozygous mice for the *Il22ra2* allele by DNA-PCR. **(E)** Transcriptional expression of *Il22ra2* in ear skin (left panel) and PLNs (right panel) in WT mice and *Il22ra2*$^{-/-}$ mice was analyzed by RT-qPCR, and expression was normalized to the *Gapdh* transcript. Data are the mean ± s.d. from three individual samples in a single experiment. **P* < .01 compared with WT mice. All data are representative of at least three independent experiments.

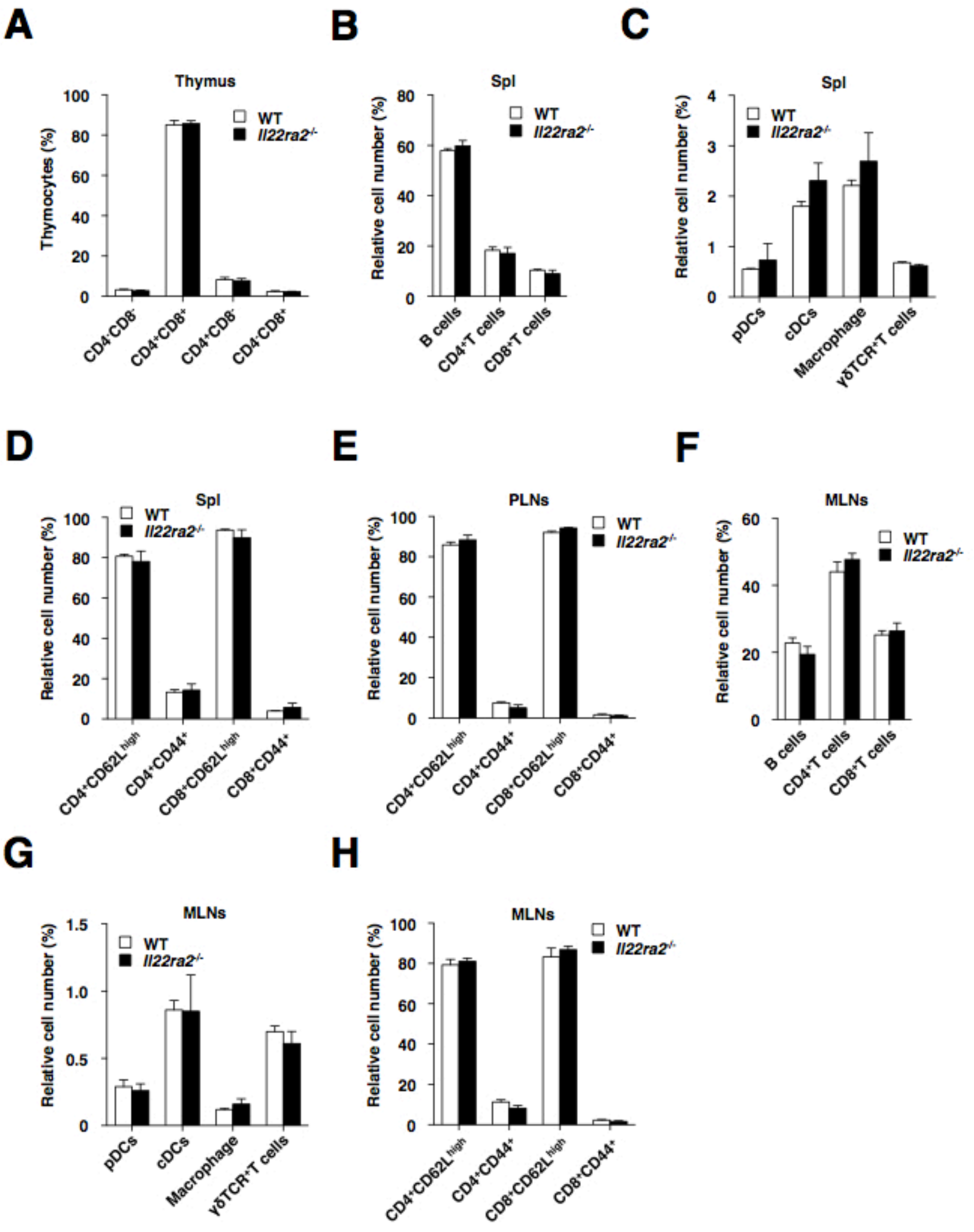

FIGURE S4. Normal constituencies of leukocytes in *Il22ra2*$^{-/-}$ mice. The frequency of the indicated leukocytes in thymus **(A)**, Spl (**B-D**), PLNs **(E)**, and MLNs **(F-H)** was analyzed by flow cytometry in WT mice (n=3) and *Il22ra2*$^{-/-}$ mice (n=3). Data are the mean ± s.d. from three individual samples in a single experiment. All data are representative of at least three independent experiments.

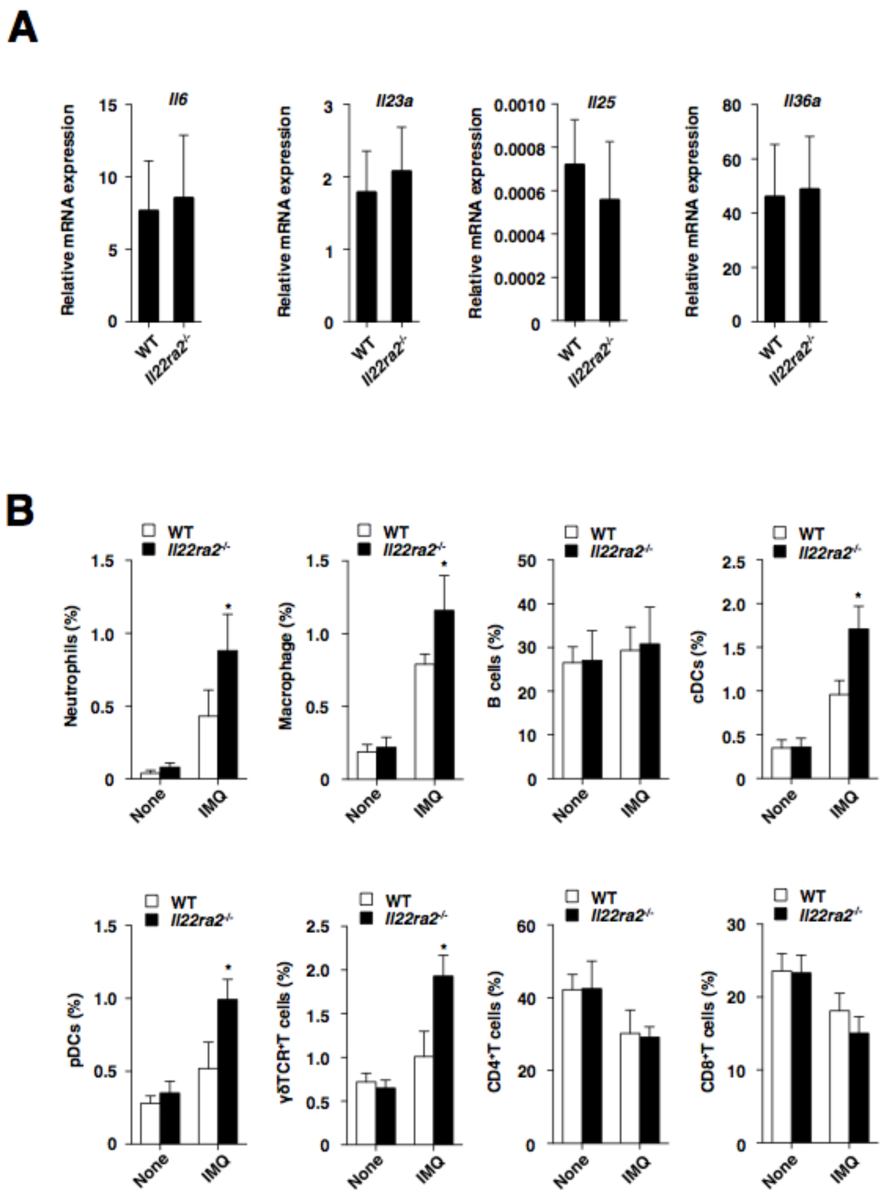

FIGURE S5. Influence of IL-22BP deficiency on the induction of skin inflammation. **(A)** Transcriptional expression of *Il6*, *Il23a*, *Il25*, and *Il36a* in ear skin was analyzed by RT-qPCR at 8 days after topical application of IMQ on the ear skin in WT mice (n=5) and *Il22ra2*$^{-/-}$ mice (n=5), and expression was normalized to the *Actb* transcript. Data are the mean ± s.d. from five individual samples in a single experiment. **(B)** The frequency of leukocytes in PLNs was analyzed by flow cytometry at days 0 and 8 after topical application of IMQ on ear skin in WT mice (n=5) and *Il22ra2*$^{-/-}$ mice (n=5). Data are the mean ± s.d. from five individual samples in a single experiment. *$P < .01$ compared with WT mice. All data are representative of at least three independent experiments.

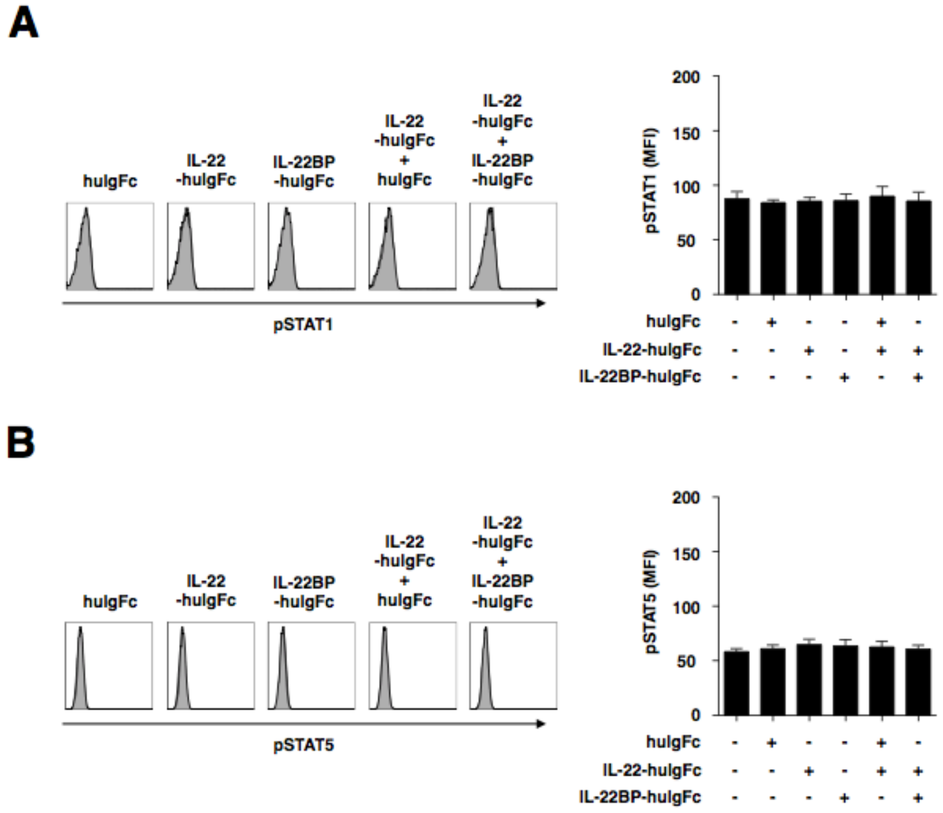

FIGURE S6. Effect of IL-22BP on the IL-22-mediated expression of pSTAT1 and pSTAT5 in keratinocytes. Keratinocytes were cultured with or without huIgFc, IL-22-huIgFc, and/or IL-22BP-huIgFc in the absence of extracellular calcium for 30 min, and the expression of pSTAT1 (**A**) and pSTAT5 (**B**) in nucleus was analyzed by flow cytometry. Data are presented as a histogram (left panel). Data are the MFI ± s.d. from three individual samples in a single experiment (right panel). All data are representative of at least three independent experiments.

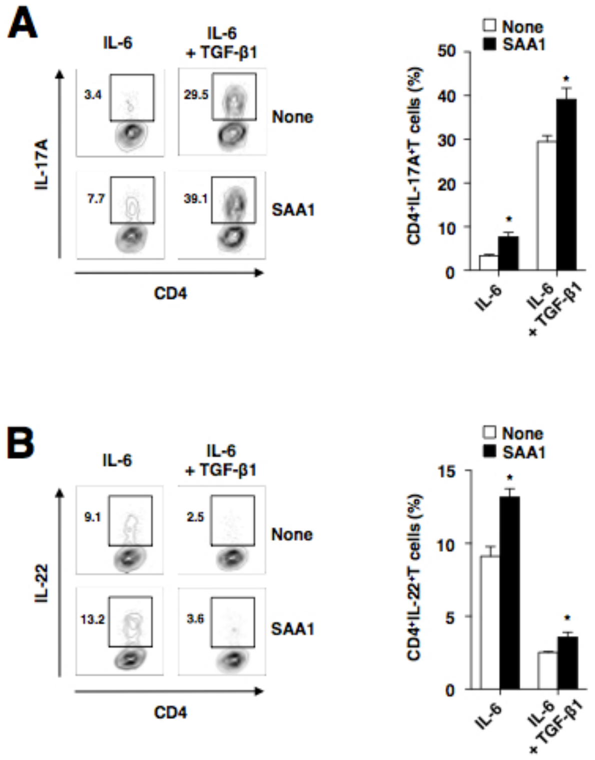

FIGURE S7. Influence of SAA1 on the generation of Th17 cells and Th22 cells. $CD4^+$ T cells were cultured with Spl cDCs in the presence or absence of SAA1 for 3 days under Th17 **(A)**- or Th22 **(B)**-polarized culture conditions. Subsequently, the frequency of IL-17A-producing cells **(A)** and IL-22-producing cells **(B)** among $CD4^+$ T cells was analyzed by flow cytometry. Data are presented as a contour plot, and numbers represent the proportion of the indicated cell populations in each gate (left panel). Data are the mean ± s.d. from three individual samples in a single experiment (right panel). *P < .01 compared with WT mice. All data are representative of at least three independent experiments.

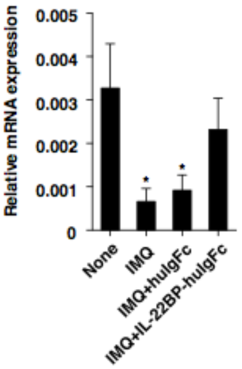

FIGURE S8. Influence of IL-22BP on the transcriptional expression of *Il25* in IMQ-induced psoriasiform dermatitis. WT mice (each n=5) that had received topical application of IMQ on the left ear skin every day for 5 days were treated with or without huIgFc or IL-22BP-huIgFc every other day. Transcriptional expression of *Il25* in ear skin were analyzed by RT-qPCR at 5 days, and expression was normalized to the *Actb* transcript. Data are the mean ± s.d. from three individual samples in a single experiment. **P* < .01 compared with untreated control. All data are representative of at least three independent experiments.
